# In Situ Synthesis of Silver Nanoparticles on Amino-Grafted Polyacrylonitrile Fiber and Its Antibacterial Activity

**DOI:** 10.1186/s11671-021-03496-0

**Published:** 2021-02-16

**Authors:** Guangyu Zhang, Yao Xiao, Qitao Yin, Jiawei Yan, Chuanfeng Zang, Huiyun Zhang

**Affiliations:** 1 National and Local Joint Engineering Research Center of Technical Fiber Composites for Safety and Health, School of Textile and Clothing, Nantong University, Nantong, 226019 People’s Republic of China; 2 Faculty of Textile Science and Technology, Shinshu University, 3-15-1, Tokida, Ueda, Nagano, 386-8567 Japan; 3 Dongfang Hospital Affiliated to Beijing University of Chinese Medicine, Beijing, 100078 People’s Republic of China

**Keywords:** Polyacrylonitrile fiber, Amino hyperbranched polymers, Ag nanoparticles, Steaming method, Antibacterial property

## Abstract

In this study, amino hyperbranched polymers (HBP)-grafted polyacrylonitrile (PAN) fiber was prepared through an amidation reaction in an autoclave. The prepared PAN-G-HBP fiber can complex Ag^+^ through amino groups of amino HBP, and in a hot steaming condition, Ag^+^ can be converted to Ag0 through the reducibility of HBP. PAN-G-HBP and Ag nanoparticles (NPs)-coated fibers were then characterized through FTIR, UV–VIS DRS, FE-SEM, EDS, XPS and antibacterial measurement. FTIR results confirmed HBP was grafted on the surface of PAN fiber. FE-SEM showed that after grafting with HBP, the average diameter of PAN fibers was amplified. EDS, XPS, and UV–VIS DRS method indicated that under hot steaming condition and with the reducibility of HBP, Ag NPs uniform coating on the PAN-G-HBP. Ag NPs-coated fibers exhibits excellent antibacterial property against *Escherichia coli* and *Staphylococcus aureus.* Even under 20 times home washing conditions, the antibacterial reduction of Ag NPs-coated PAN fiber can achieved more than 98.94%.

**Supplementary Information:**

The online version contains supplementary material available at 10.1186/s11671-021-03496-0.

## Introduction

Polyacrylonitrile (PAN) fiber, obtained by free radical polymerization of monomer acrylonitrile, has excellent resistance to weather, sun, acid, and oxidant [1–3]. Generally, PAN fiber is used to replace or mix wool fabric and is suitable for interior decoration cloth, such as curtains. PAN fiber products are fluffy, soft, with an ultra-fine diameter and a large specific surface area [4–6]. This product is limited in industrial use due to the lack of functional groups in the molecular structure of PAN [7]. The cyano-groups of PAN fiber can be easily transformed into various active groups, such as amination, amidoximation, and sulfonation, then the groups can be further grafted to obtain the functional PAN fibers and expand its application in various fields [8–11]. Wang et al. [12] investigated the modification of PAN fiber by hyperbranched polyethylenimine (HPEI) through water-mediated hydrolysis and amidation reaction in an autoclave.The obtained fibers could be successfully used as supporters and stabilizers in the preparation of small-sized Au nanoparticles (NPs). Ju et al. [13] investigated the polyamidoamine grown on the surface of the PAN fiber and found that the treated fiber can be extracted from seawater through adsorption of uranium. In these reports, HPEI are very expensive polymers, controlling the graft of the PAN fibers are always difficult and needs many steps.

Silver is a widely used material and has been proven to be effective against bacteria, fungi, and viruses. The fiber containing Ag NPs has been prepared for a variety of applications, including biotextiles, wound dressings, biological protection materials, sportswear, and so on. Studies attempted to improve the combination of Ag NPs and fiber [14]. Many reducing agents, such as glucose, sodium borohydride (NaBH_4_), and polyphenol, were used for the reduction of Ag^+^ to obtain Ag NPs. The coating strategy on fibers mainly depends on solution-based assembly technology, which mainly includes pad dry curing, spraying, in-situ deposition, and sol–gel coating. Moreover, polyvinyl acetate, polyurethane resin, and polyacrylic esters were essential to fix the silver NPs on the fibers [15]. Therefore, the application of the Ag Nps on the surface of fibers is often difficult and requires many steps [16].

In our previous studies, an amino hyperbranched polymer (HBP) containing several amino groups and spherical three-dimensional structure with inner nano-cavities was synthesized [17], the terminated amino group can easily produce chemical adsorption with heavy metal particles and its nano-cavities was applied to the control synthesis of silver NPs and ZnO NPs [18–22].

In this work, Ag NPs-coated PAN fibers were prepared to enhance the antibacterial properties of PAN fiber. First, PAN fibers were used as the matrix, and amino HBP was grafted onto PAN fibers to prepare polyamine-modified PAN fibers. Then, in the coating process, HBPs were used as a complexing agent to capture the Ag^+^ in aqueous solution, and in a hot steaming condition, Ag^+^ was reduced to Ag^0^ by an amino group. Amino HBP can entrap Ag NPs in the confined internal cavity and prevent them from further aggregation due to the three-dimensional spherical structure and internal nanocavity. Compared with the reported methods, the synthetic process of HBP is simple and with low price. In the coating process, HBP as a reducing agent and binder to fix the Ag NPs on the surface of PAN fibers to provide antimicrobial properties, no other auxiliaries were used.

## Methods

### Materials

PAN fibers with a length of 2–3 cm were obtained from Suzhou Weiyuan in China. The copolymerization of acrylonitrile (95 wt%), methyl acrylate, trace sodium styrene sulfonate (5 wt%), and amino HBP were prepared as described in our paper [17]. AgNO_3_ (analytically pure) and BasO_4_ (spectral purity) were purchased from Guoyao Chemical Reagent, China. *Staphylococcus aureus* (*S. aureus*) (ATCC 6538) and *Escherichia coli* (*E.coil*) (ATCC 8099) were obtained from the Shanghai Luwei Technology Co., Ltd. (China).

### Synthesis of PAN-G-HBP Fiber

HBP solutions of 20 mL of 4, 8, 16, and 24 g/L were prepared in the autoclave, and 1-g PAN fibers were added to the amino HBP solution. The mixtures were sealed up in autoclave at 120 °C for 2 h. After cooling down, the PAN fiber was washed by water and ethyl alcohol separately. The fiber was then dried at 80 °C for 60 min to obtain the PAN-G-HBP fiber.

### Preparation of Ag NPs-Coated PAN-G-HBP Fiber

A certain amount of PAN-G-HBP fibers placed in a 0.1–0.5-mM AgNO_3_ aqueous solution for 60 min with a liquor ratio of 1:30. Subsequently, the PAN-G-HBP fibers were steamed (100 °C) for 30 min using a steam engine (BTZS10A, China). Then, the fibers were washed by deionized water and dried at 60 °C to produce the Ag NPs-coated PAN fibers.

### Measurements

Fourier-transform infrared (FTIR) analysis spectra were performed using a Nicolet 5700 FTIR spectrophotometer (Thermo Electron Corporation, USA). The surface morphology of fibers was characterized using a Field Emission Scanning Electron Microscope (FE-SEM) (Scios DualBeam, Czechia) and energy dispersive spectroscopy (EDS) (Carl Zeiss, EVO 15, Oberkochen, Germany). The tensile properties of fibers were studied using the fiber testing machine (ZEL-A-2, Shanghai, China). Ultraviolet–visible diffuse reflection spectroscopy (UV–vis DRS) of Ag NPs-coated PAN fiber was carried out through UV-2550 (Shimadzu, Japan), with BaSO_4_ powers as a reference. X-ray photoelectron spectroscopy (XPS) analyses were carried out using an XSAM 800 electron spectrometer (Kratos, UK). The Ag content in the PAN fibers was measured using a Vista MPX Inductively Coupled Plasma Atomic Emission Spectrometer (ICP-AES) (Varian, USA). The Ag content was calculated using Eq. (1).

1 $${\text{Ag}}\;{\text{contents}}\;\left( {{\text{mg}}/{\text{g}}} \right) = \frac{C*V}{M},$$

where *C* (mg/L) is the concentration of Ag in the solution, and *V* (L) and *M* (mg) represent the volume of solution and weight of fiber, respectively.

The antimicrobial activity of PAN, PAN-G-HBP, and Ag NPs-coated PAN fibers was tested by studying the growth kinetics of *S. aureus* and *E. coli* [23]. Fibers at 0.8 g were placed separately in the bacterial suspension of *S. aureus* and *E. coli*. They were cultured sealed in the oscillator at 37 °C for 6 h and sampled once every 30 min. The optical density of the bacterial suspension at 546 nm was measured using an ultraviolet–visible (UV–vis) spectrophotometer (UV-3010, Hitachi, Japan). The antimicrobial rate of the above fibers was tested against *E. coli and S. aureus* through the shaking flask method following GB/T20944.3-2008 (China) [24]. The washing durability of Ag NPs coated PAN fiber was evaluated according GB/T 20944.3-2008 (China). The fiber was put in a stainless-steel container containing 150 mL of 0.2% (w/v) AATCC WOB standard detergent solution and 10 steel balls for 45 min, the temperature was 40 °C. This process was equivalent to five washing cycles for home washing. The Ag contents and antibacterial activity afte5 and 20 washing cycles was determined.

## Results and Discussion

### Preparation and Characterization of Amino-Grafted PAN Fiber

**Scheme 1:**
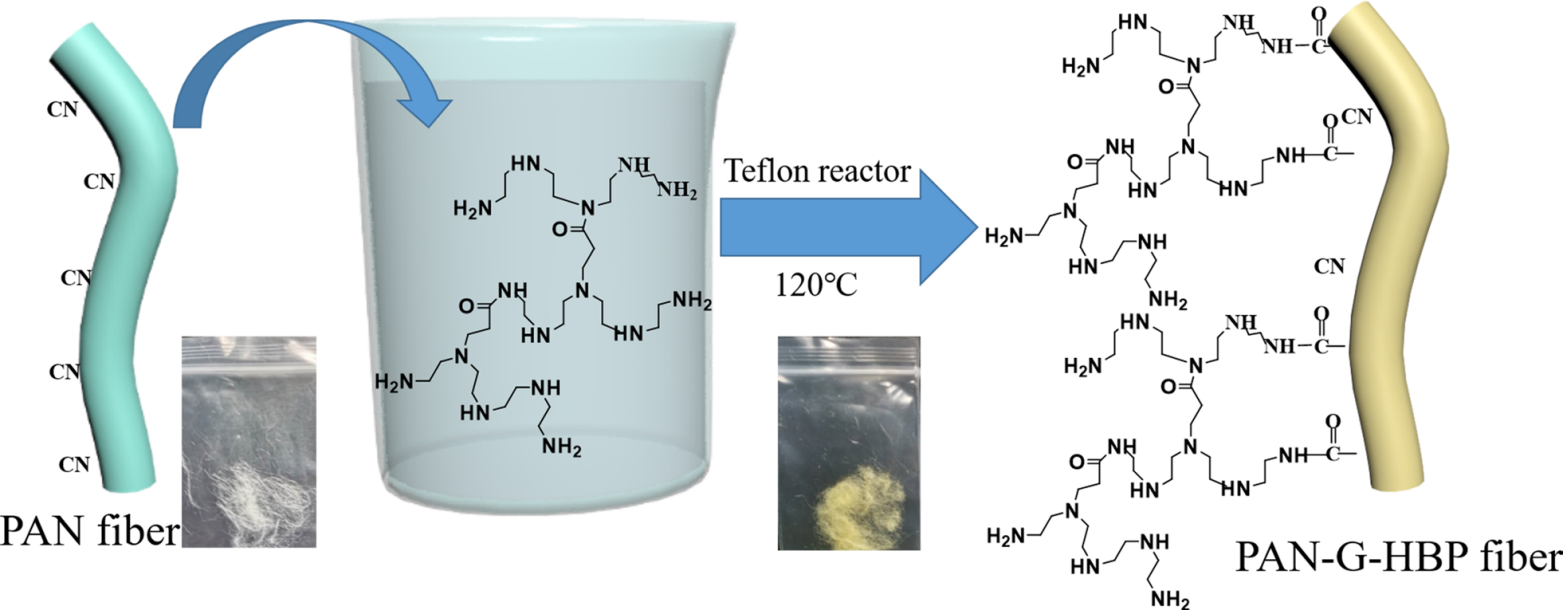
Preparation of PAN-G-HBP fiber

The PAN fiber is corrosion and resistant, with excellent mechanical strength and stability. In addition, the fiber is rich in cyano groups, which are easily converted into various functional parts (carboxyl, amide, or amidoxime groups) [25]. The nitrile groups on the surface of PAN fibers were hydrolyzed and then amidated with amino HBP (Scheme 1) to obtain PAN-G-HBP fibers. The PAN fiber was graft-modified with amino HBP at a concentration of 0, 8, 16, and 24 g/L. The amino HBP aqueous solution is alkaline due to the cationic properties of the amino group. If the concentration is high, then the alkaline is strong. In the alkaline solution at a high temperature and pressure condition, the PAN fiber portion-CN is hydrolyzed to form a COO– group. Then, COO– reacts with the terminal amino group of the amino HBP to form a –CO–NH– group, and in this reaction, the white PAN fiber gradually changed into light yellow. Thus, the amino HBP was successfully grafted on the surface of PAN fibers [26, 27].

**Fig. 1:**
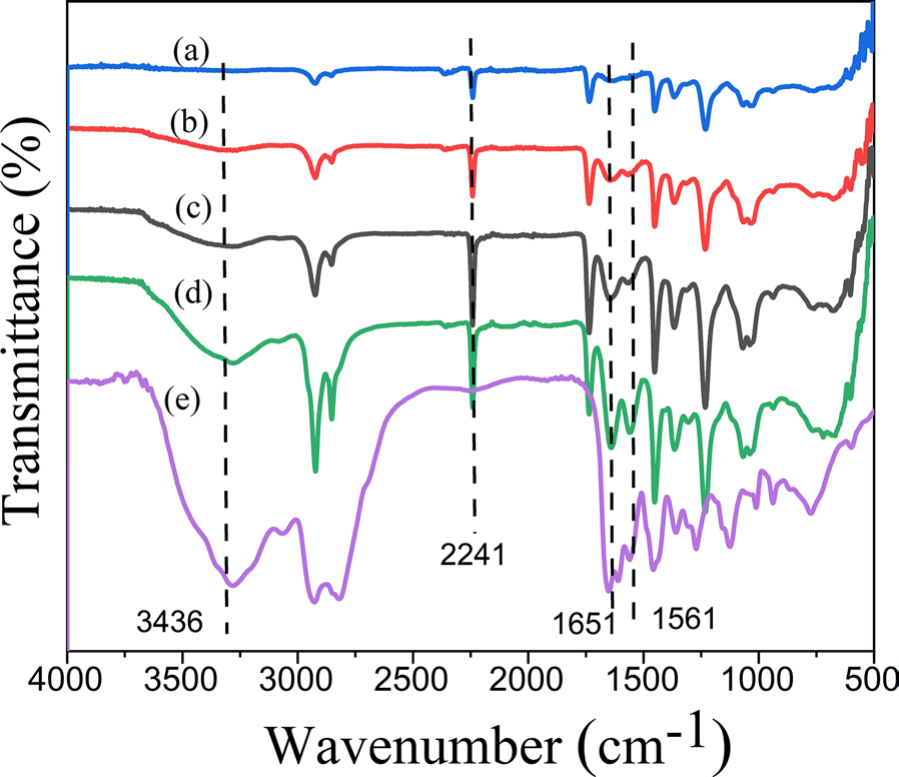
FT-IR of PAN **a** grated with **b** 8 g/L, **c** 16 g/L, and **d** 24 g/L amino HBP **e** HBP

The grafted PAN fiber was characterized by the FTIR method to further verify the group changes in the reaction. Compared with the FTIR spectrum of pure PAN fiber (Fig. 1a), many new characteristic absorption peaks appeared in the FTIR spectrum of PAN-G-HBP (Fig. 1b–d). For instance, the absorption peak is at approximately 3400 cm^−1^, which is the characteristic of the N–H stretching bond frequency of the primary, secondary amine, and amide groups of the HBP. Moreover, the C=O stretch bond frequency of the amide group was absorbed at 1651 cm^−1^ [22, 28, 29].The strong C=O asymmetric tensile bonding frequency of COO– can be observed at 1563 cm^−1^, which overlaps with N–H deformation and C–N tensile vibration. According to the spectrum of HBP (Fig. 1e), the new exhibited absorption peaks of PAN fiber at 3436, 1651 and 1563 cm^−1^ can be attributed to the characteristic absorption of HBP [30]. All these results confirmed that amino HBP was successfully grafted onto PANF. The strong absorption at 2242 cm^−1^, which is characteristic of C≡N stretch bond frequency, also exists in the spectrum of PAN-G-HBP. This finding indicates that only certain nitrile groups of PAN participate in the reaction. The reason for this can be attributed to the high molecular chain regularity of polyacrylonitrile, the grafting reaction mainly occurs in the amorphous region. After grafting with HBP, larger volume steric hindrance is produced, making it difficult for HBP to penetrate into the inner part of the fiber [12, 29].

**Fig. 2:**
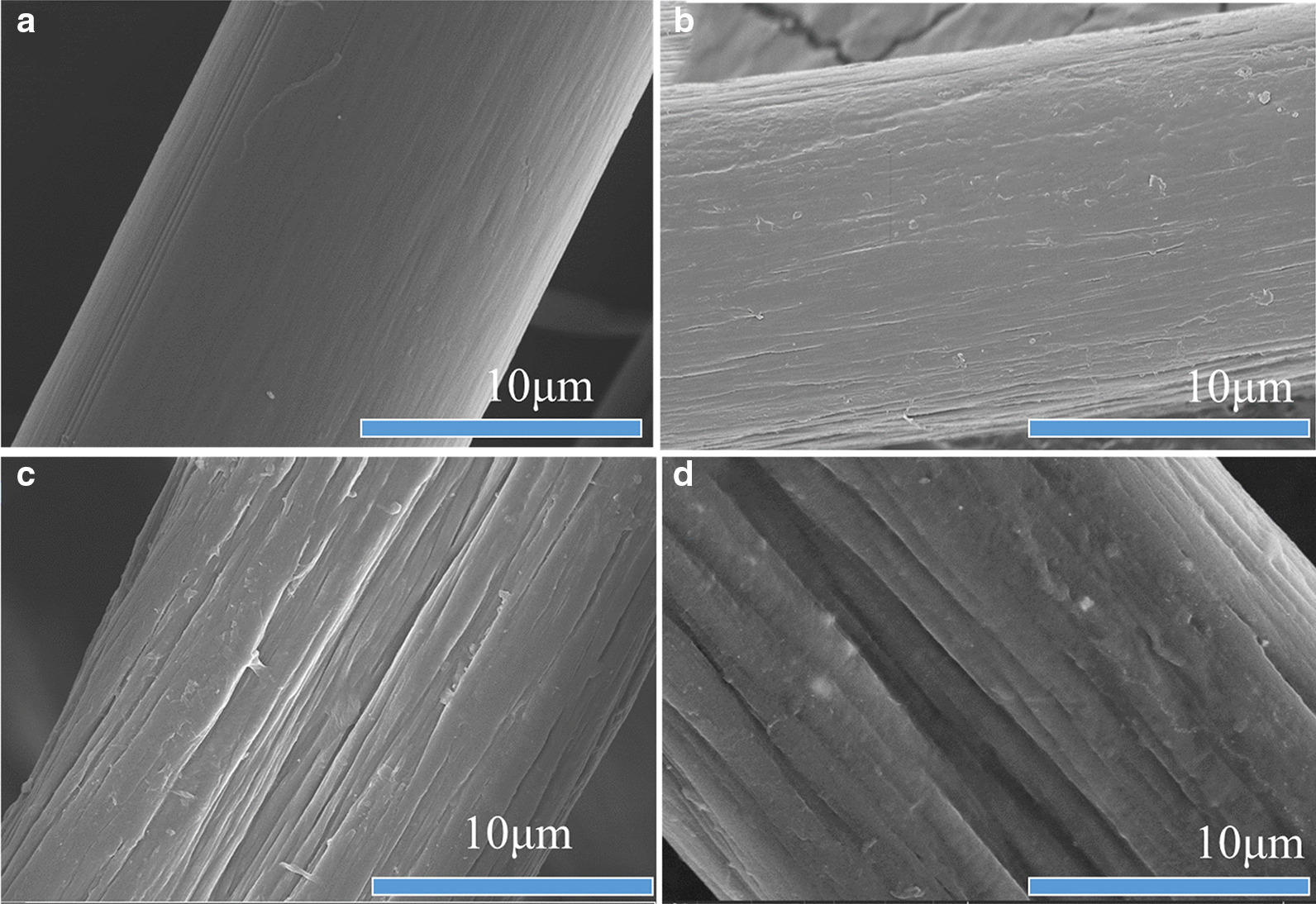
SEM images of **a** pure PAN grafted with **b** 8 g/L, **c** 16 g/L, and **d** 24 g/L amino HBP

The PAN and the PAN-G-HBP fiber were also characterized by FE-SEM. Figure 2a shows that the surface of the original fiber is smooth, the structure is dense and uniform, and longitudinal grooves are very shallow [31, 32]. After grafting with amino HBP, the fiber surface morphology (Fig. 2b–d) becomes rough and uneven and has a hollow core structure. If the amino HBP is high, then the diameter of the PAN fibers is large. As the degree of amination continues to increase, the surface morphology becomes increasingly rough, the dents continue to deepen and widen, the folds are evident, and the degree of damage continues to increase. The reason for this is that amination modification mainly occurs on the surface of the fiber, after the amination modification, the volume of HBP is large and occupies more space of the modified fiber, and the bond between the macromolecular chains of the PAN fiber becomes looser, making the space crowded [33].

Additional file 1: Fig. S1 shows the influence of HBP concentration on fiber weight gain. As the concentration of amino HBP increases, the number of amine groups increases. The kinetics shows that the weight gain rate increases with the increase of amino HBP concentration. Additional file 1: Fig. S2 shows the breaking strength of the PAN fiber grafting with different amino HBP concentrations. With the increase of the amino HBP concentration, the breaking strength of PAN fiber descends. The reason for this can be ascribed that the amination modification mainly occurs on the surface of the fiber. After the amination modification, the volume of amino HBP occupies additional space of the modified fiber, and a part of the crystallized area was destroyed, leading to the reduction in strength of the fiber [10, 12]. Hence, we choose 16 g/L amino HBP for the treatment of the PAN fiber to achieve the balance between the breaking strength and grafting ratio.

### Preparation of Ag NPs-Coated PAN Fiber

**Scheme 2:**
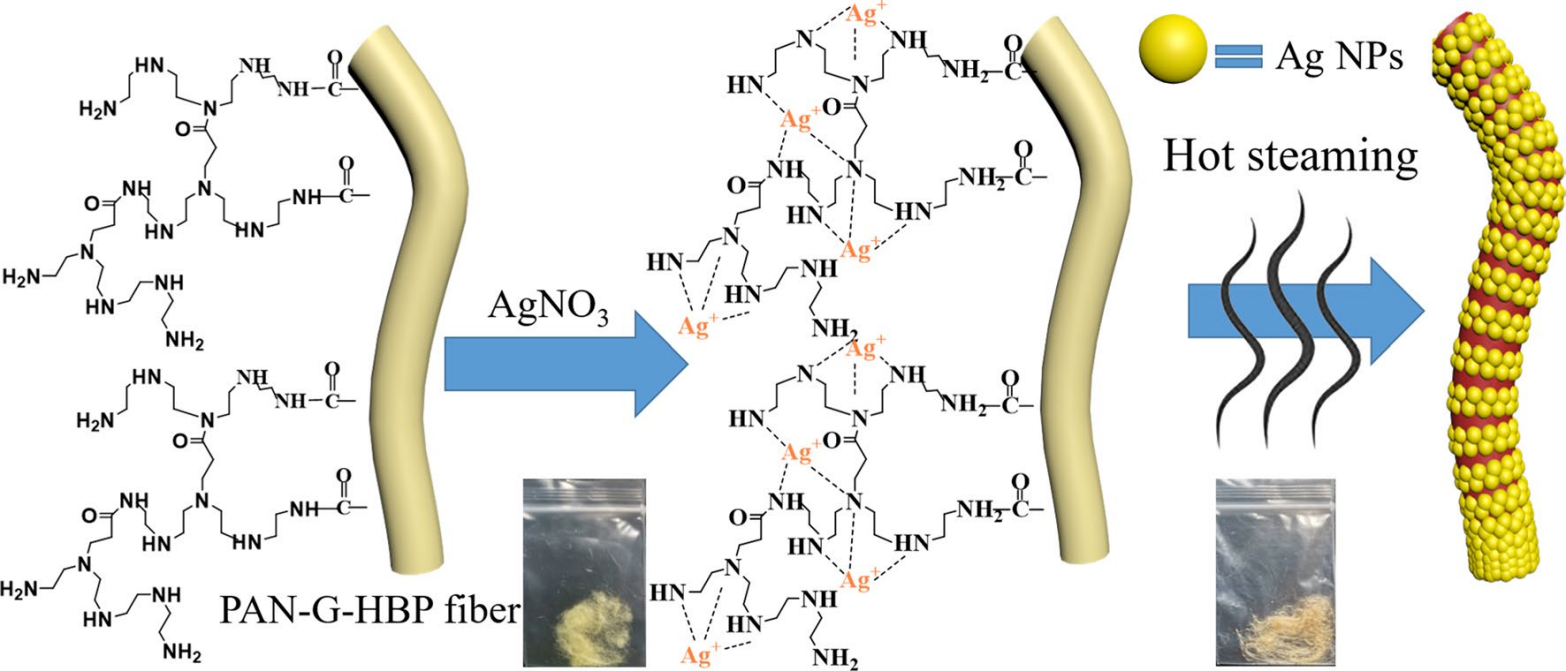
Ag NPs-coated on PAN-G-HBP fiber

Scheme 2 depicts the principle of dispersed Ag NPs on PAN fiber. Amino HBP was characterized by a three-dimensional structure and contained a large number of amino groups and terminal primary amino groups, which are suitable for complex metal ion in water [13, 34]. Under a high-temperature condition, amino groups can reduce Ag^+^ to form a sliver colloid without any additional reductants. These amine groups on the PAN fibers can attract silver ions and provide an electron source for the reduction process. In this reaction, HBP plays an important role in reducing silver ions (Ag^+^) to form silver NPs (Ag^0^), as an efficient self-reducing agent and preventing the agglomeration of NPs as a stabilizer. The Ag NPs are confined in the interior of the polymers, and their growth will be physically restricted by the meshes [16]. Hence, the size and size distribution can be effectively controlled. When the reaction is completed, the yellow fibers gradually changed into brown.

### Antibacterial Properties of Ag NPs-Coated PAN Fiber

PAN-G-HBP samples were immersed in 0.1, 0.2, 0.3, 0.4, and 0.5 mM AgNO_3_ solution, and marked with a, b, c, d, and e, respectively, to provide PAN fabrics with antibacterial properties. After treated in a hot steaming condition (100 °C) for 30 min, Ag NPs were coated on the fiber. Additional file 1: Table S1 shows the silver content and antibacterial properties against *E. coli* and *S. aureus* of the samples. The PAN fiber did not show antibacterial activity against *S. aureus* or *E. coli* indicating that the PAN fiber alone is not sufficient to inhibit the growth of bacteria. Attributed to its cationic properties of the amino group, the PAN-G-HBP fibers show certain antibacterial activity [35]. This finding indicates that amino HBP can potentially enhance the antibacterial properties of the PAN fiber. By contrast, the Ag NPs-coated PAN fiber exhibits excellent antibacterial activity even with Ag contents at 110 mg/kg. When the concentration of the silver reaches to 270 mg/kg, cells hardly survive on the PAN fiber. The laundering durability of the Ag NPS-coated PAN fibers are very import factors to consider. After washing 5 times and 20 times, the silver content and antibacterial activity of the Ag-coated PAN were measured and the results are shown in Additional file 1: Table S2. As the washing cycle increases, the silver content and antibacterial activity of the Ag-coated PAN decreases. After 20 washing cycles, the fiber still showed a bacterial reduction of 99.11% and 98.94% for *S. aureus* and *E. coli*, respectively. The excellent durability of Ag NPs on PAN fibers are attributed to the unique chemical and physical properties of HBP, it can trap silver ions in the narrow internal cavity, and prevent them from further gathering through electrostatic and steric hindrance effects [24]. We choose sample c (treated by 0.3-mM AgNO_3_) for further characterization.

Growth kinetics of *E. coli* and *S. aureus* in the presence of PAN, PAN-G-HBP, and Ag NPs-coated PAN fibers (Ag content of approximately 270 mg/kg) were studied to evaluate the antibacterial kinetics of the Ag NPs-coated PAN fiber. Figure 6 shows the results. The optical density of *E. coli* and *S. aureus* bacterial suspension at 546 nm began to increase after 0.5 h. In the presence of amino HBP, the optical density of the bacterial suspension of *E. coli* and *S. aureus* began to increase in 1 h. At approximately 6 h, the absorbance of bacterial suspension was the same as that of the blank sample. This result is because the positive amino inhibits the growth of bacteria at the beginning of the culture. As the culture time increases, its inhibitory effect gradually disappears [23]. On the contrary, the optical density of the bacterial suspension of *E. coli* and *S. aureus* never increased during the entire experimental period in the presence of Ag NPs-coated PAN fiber. Therefore, the Ag NPs-coated PAN fiber not only inhibits the growth and reproduction of bacteria but also plays a bactericidal effect to a certain extent.

### Characterization of Ag NPs-Coated PAN Fiber

**Fig. 3:**
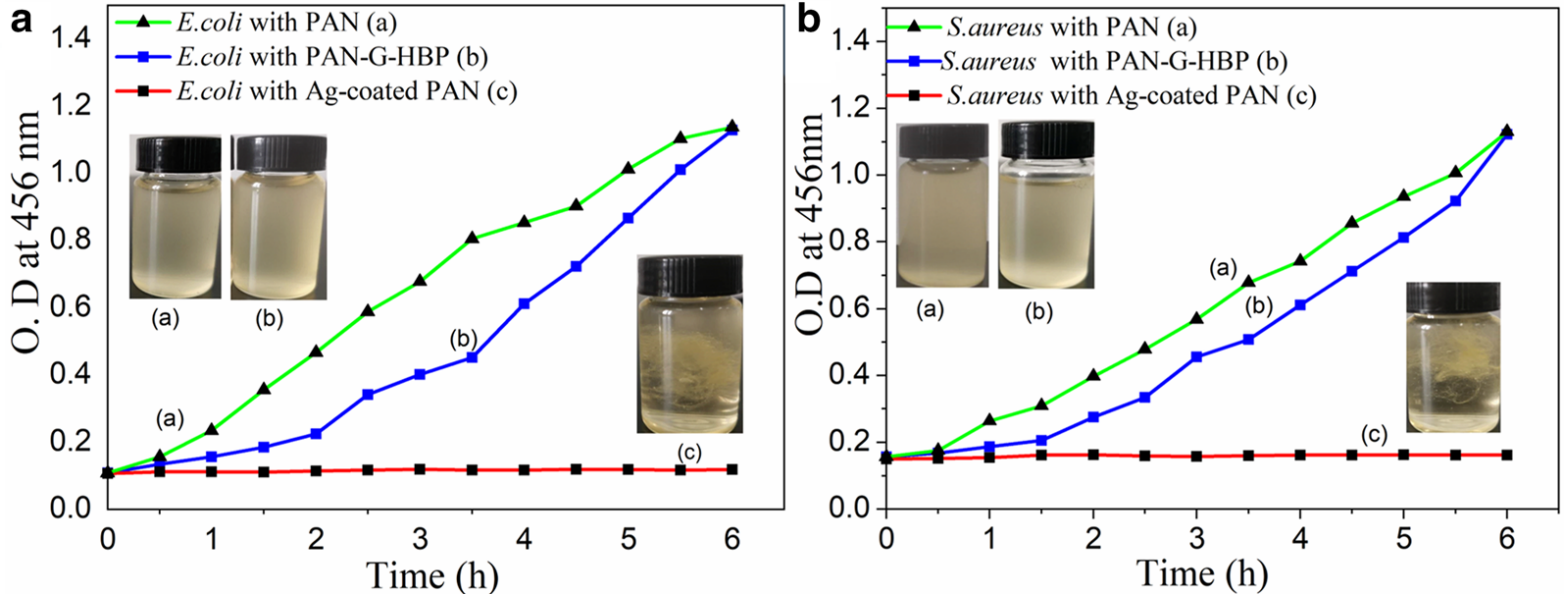
Growth kinetics of **a** *E. coli* and **b** *S. aureus* in the presence of PAN, PAN-G-HBP, and Ag-coated fibers

**Fig. 4:**
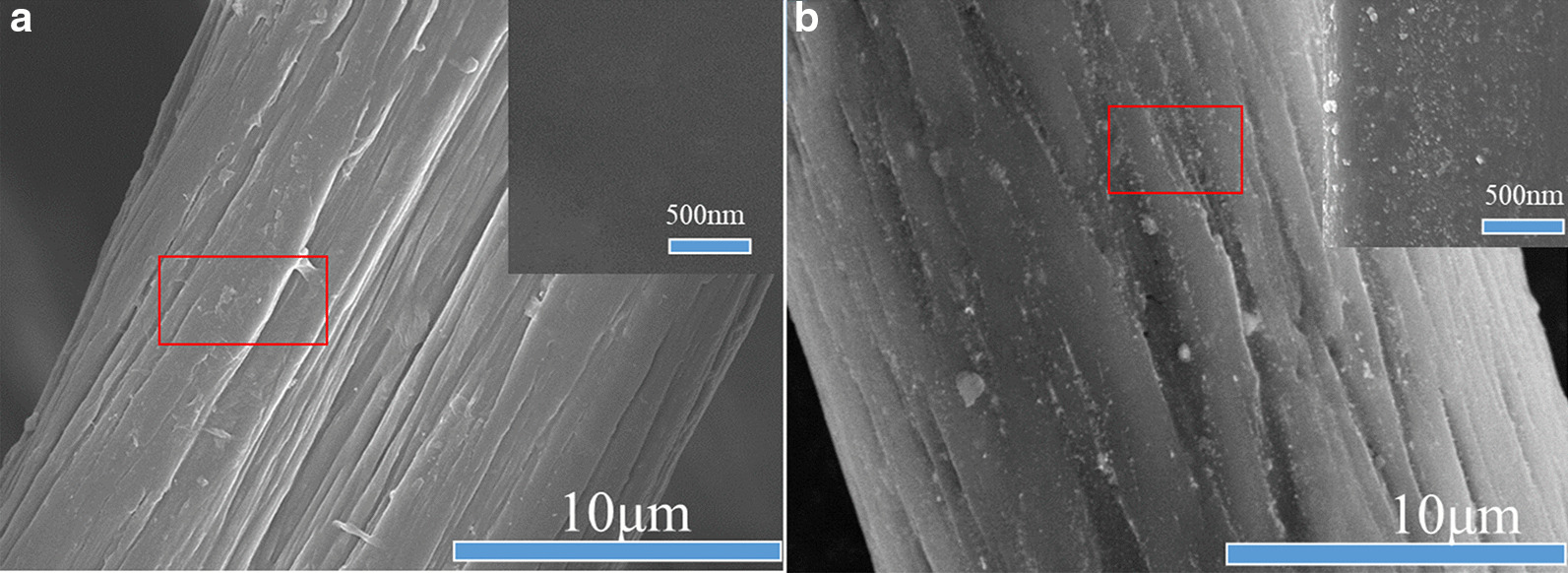
FESEM images of **a** PAN-G-HBP and **b** Ag NPs-coated fibers

The surface morphology of PAN-G-HBP and Ag NPs-coated PAN fibers was further investigated through FESEM. Figure 3 depicts PAN-G-HBP and Ag NPs-coated PAN fibers, showing an evident distinction between the two fibers. The surface of the PAN-G-HBP fiber was flat and smooth (Fig. 4a), whereas many white spots can be found on the PAN fiber after treated with Ag^+^, and the white spots were uniformly dispersed on PAN fiber surface.

**Fig. 5:**
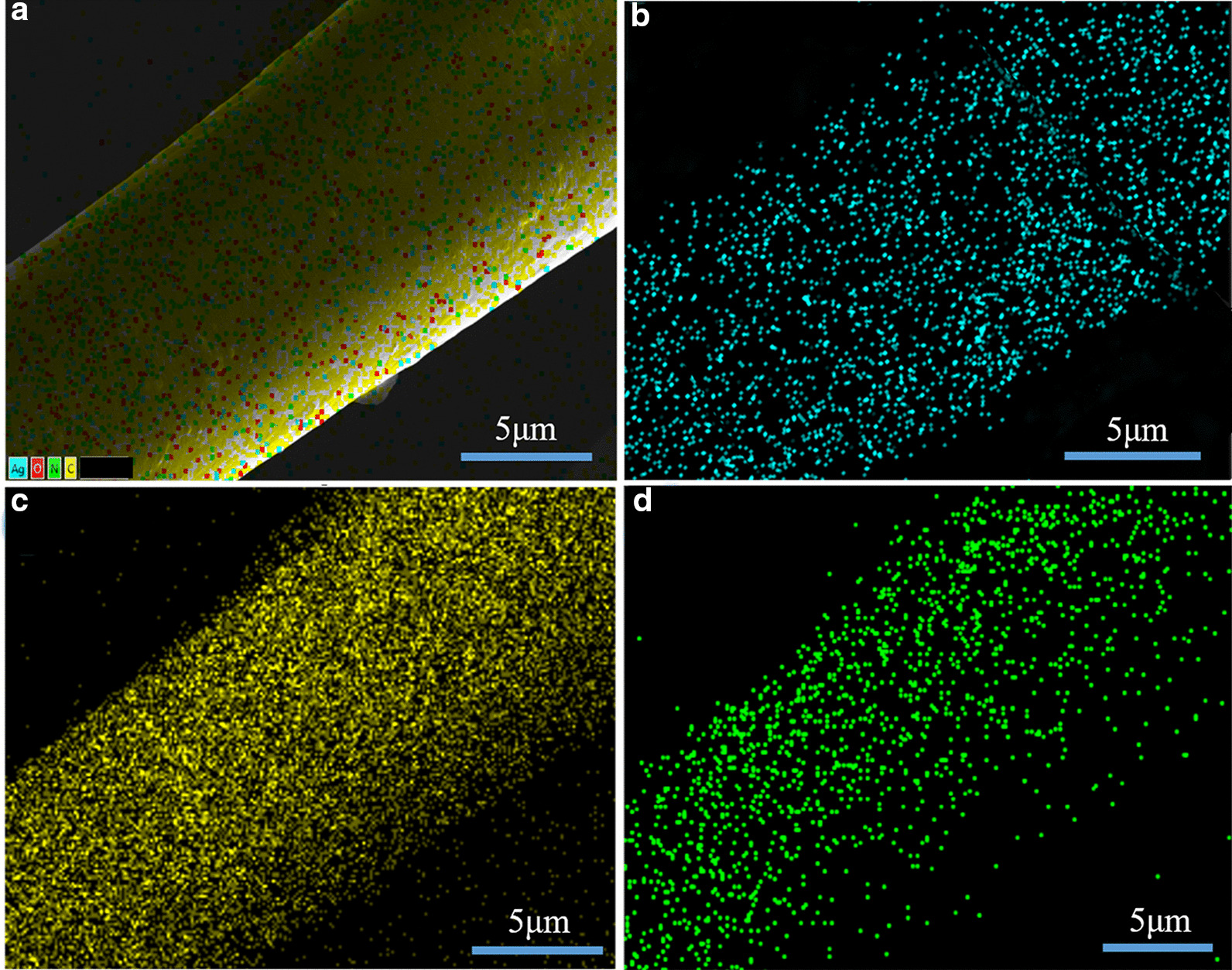
EDS mapping images of elements on **a** a PAN fiber with **b** Ag, **c** C, and **d** N

The chemical characteristics of the Ag NPs-treated PAN-G-HBP fiber were further examined through the EDS analysis of elements C, O, and Ag to confirm whether the white spots were sliver. Figure 5a and Additional file 1: S3 show that an additional Ag element was found in the PAN fibers which may be ascribed to the attachment of Ag NPs to the PAN-G-HBP fibers. Figure 5b–d depicts C and N including the even distribution Ag element on the PAN fiber surface. Notably, Ag was distributed evenly across the PAN fiber surfaces, and the result was in good agreement with the FESEM measurements.

**Fig. 6:**
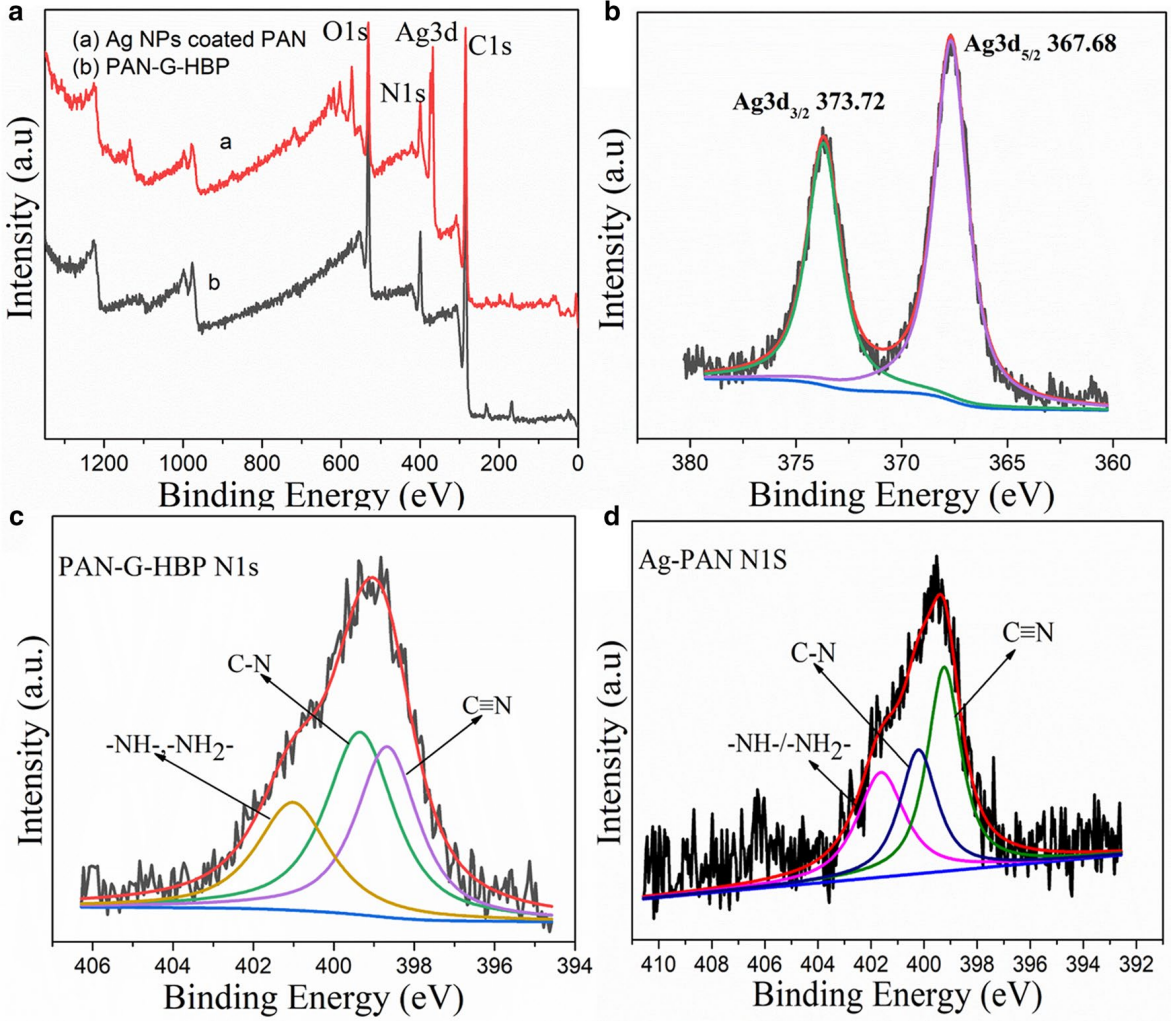
**a** High-resolution XPS spectra, **b** Ag 3*d*, **c** N1*s* for PAN-G-HBP fiber, and **d** N1*s* for Ag-coated PAN fiber

XPS and UV–vis DRS analyses of the Ag NPs-coated PAN fibers were conducted to further investigate the Ag NPs coating process. Figure 6a shows that PAN-G-HBP fibers displayed peaks of O1*s*, N1*s*, and C1*s*. New Ag3*d* peaks at 373 eV were observed after treated with Ag^+^, indicating the coating of Ag element on the PAN fiber. Ag NPs are easily oxidized when exposed to the air without good protection. In Fig. 6b, two peaks at 367.68 and 373.72 eV can be attributed to Ag3*d*3/2 and Ag3*d*5/2 of metallic Ag NPs respectively, indicating a good protection of Ag NPs by amino HBP [36]. The core energy levels of N1*s* were also investigated to further investigate the amide bond change in the coating process, as shown in Fig. 6c, d. The N1*s* spectrum of the material forms three peaks at approximately 399 eV, belonging to –NH_2_/–NH–, –C–N–, and C≡N. Figure 6a, c, d shows that the intensities of N1*s* decreased, and the peaks of N1*s* shifted to higher energy values [13, 33].The results confirmed the participation of N-containing groups in the coating process. The UV–vis DRS spectrum of PAN fibers has a broad UV absorption peak at 409 nm (Additional file 1: Fig. S4) owing to the absorption of Ag NPs [24]. This finding indicates the existence of Ag NPs on the surface of the PAN fiber.

## Conclusion

As cyano-groups of PAN fiber can be transformed into active groups, in this research the PAN fiber grafted with amino HBP through an amidation reaction in an autoclave. The obtained PAN-G-HBP has a large diameter and contained several amino groups due to the reaction between PAN and amino HBP. Amino groups on the PAN fiber could effectively complex Ag^+^ in an aqueous solution and under a high steaming condition, the Ag^+^ can convert to Ag^0^ NPs through the reducibility and protection of amino HBP. The measurements confirmed the Ag NPs were synthetized and uniformly distributed on the surface of PAN fiber. The Ag contents of the PAN fiber at 270 mg/kg show good antibacterial and washable properties. Ag NPs-coated PAN fiber not only inhibits the growth and reproduction of bacteria but also plays a bactericidal effect to a certain extent.

## Supplementary Information

**Additional file 1: Fig. S1**. the weight gain rate of PAN fibers grafted with different concentration of HBP. **Fig.S2**. Breaking strength of PAN fibersgrafted with different concentration of HBP. **Table S1**. Antibacterial activity of PAN and Ag NPs coated PAN fiber. **Table S2**. Laundering durability of AgNPs coated PAN fiber. **Fig. S3**. EDS analyze of Ag NPs-coated PAN fiber (Ag contents 270mg/kg). **Fig. S4**. UV-vis DRS of Ag NPs-coated PAN fiber(Ag contents 270mg/kg).

## Data Availability

The datasets supporting the conclusions of this article are included within the article.

## Abbreviations

HBP: Hyperbranched polymer
PAN: Polyacrylonitrile
NPs: Nanoparticles
PAN-G-HBP: Hyperbranched polymer grafted Polyacrylonitrile
FTIR: Fourier transform infrared spectrometer
UV–VIS DRS: Ultraviolet–visible diffuse-reflectance spectrum
FE-SEM: Field emission scanning electron microscope
EDS: Electronic differential system
XPS: X-ray photo-electronic spectroscopy
*S. aureus*: *Staphylococcus aureus*
*E. coli*: *Escherichia coli*
HPEI: Polyethylenimine
ICP-AES: Inductively coupled plasma atomic emission spectrometer
