# Supplementary material for: In Situ Synthesis of Silver Nanoparticles on Amino-Grafted Polyacrylonitrile Fiber and Its Antibacterial Activity
Source: Nanoscale Res Lett. 2021 Feb 16;16:36. doi: 10.1186/s11671-021-03496-0 (PMC7886948; doi:10.1186/s11671-021-03496-0)
Supplement: Supplementary file 1 — Additional file 1: Fig. S1. the weight gain rate of PAN fibers grafted with different concentration of HBP. Fig.S2. Breaking strength of PAN fibersgrafted with different concentration of HBP. Table S1. Antibacterial activity of PAN and Ag NPs coated PAN fiber. Table S2. Laundering durability of AgNPs coated PAN fiber. Fig. S3. EDS analyze of Ag NPs-coated PAN fiber (Ag contents 270mg/kg). Fig. S4. UV-vis DRS of Ag NPs-coated PAN fiber(Ag contents 270mg/kg). [file 11671_2021_3496_MOESM1_ESM.docx]

In situ synthesis of sliver nanoparticles on amino grafted polyacrylonitrile fiber and its antibacterial activity

Guangyu Zhang^1^, Yao Xiao^1^, Qitao Yin ^1^, Jiawei Yan^2^, Chuanfeng Zang^1*^, Huiyun Zhang^3*^

1) National & Local Joint Engineering Research Center of Technical Fiber Composites for Safety and Health, School of Textile and Clothing, Nantong University, Nantong 226019, P. R. China

2) Faculty of Textile Science and Technology, Shinshu University, 3-15-1, Tokida, Ueda, Nagano 386-8567, Japan

3) Dongfang Hospital Affiliated to Beijing University of Chinese Medicine,Beijing 100078, China

E-mail: Chuanfeng Zang ([zang.cf@ntu.edu.cn](mailto:zang.cf@ntu.edu.cn)), Huiyun Zhang (tataandvv@163.com)

**Experimental**

The weight gain rate of HBP onto the PAN Fiber was calculated from equation (1):

Weight gain=[(W-W_0_)/W_0_]×100%（1）

Where W_0_ is the weight of the PAN Fiber, and W is the weight of the PAN-G-HBP fiber.





Fig.S1 the weight gain rate of PAN fibers grafted with different concentration of HBP





Fig.S2 Breaking strength of PAN fibers grafted with different concentration of HBP

Table S1. Antibacterial activity of PAN and Ag NPs coated PAN fiber

| Sample | Sliver content (mg/kg) | *S. aureus* | | *E. coli* | | |
| --- | --- | --- | --- | --- | --- | --- |
|  |  | Surviving cells (CFU/mL) | Reduction (%) | Surviving cells (CFU/mL) | | Reduction (%) |
| PAN | - | 1.38×10^6^ | - | 3.1×10^5^ | - | |
| PAN-G-HBP | - | 9.6×10^5^ | 30.43 | 2.01×10^5^ | 35.16 | |
| a | 110 | 1.62×10^4^ | 88.26 | 3.9×10^4^ | 87.42 | |
| b | 180 | 1.52×10^4^ | 98.9 | 7.3×10^3^ | 97.65 | |
| c | 270 | ＜10 | 99.99 | ＜10 | 99.99 | |
| d | 340.06 | ＜10 | 99.99 | ＜10 | 99.99 | |
| e | 450.07 | ＜10 | 99.99 | ＜10 | 99.99 | |

Table S2. Laundering durability of Ag NPs coated PAN fiber

| Sample | Sliver  Content (mg/kg) | *S. aureus* | | *E. coli* | | |  |
| --- | --- | --- | --- | --- | --- | --- | --- |
|  |  | Surviving cells (CFU/mL) | Reduction (%) | Surviving cells (CFU/mL) | | Reduction (%) | |
| PAN | - | 8.5×10^6^ | - | 1.23×10^5^ |  | |  |
| PAN-G-HBP | 282 | ＜10 | 99.99 | ＜10 | 99.99 | |  |
| 5 times washing | 251 | ＜10 | 99.99 | ＜10 | 99.99 | |  |
| 20 times washing | 207 | 7.6×10^4^ | 99.11 | 1.3×10^3^ | 98.94 | |  |





Fig. S3 EDS analyze of Ag NPs-coated PAN fiber (Ag contents 270mg/kg)





Fig. S4 UV-vis DRS of Ag NPs-coated PAN fiber (Ag contents 270mg/kg)
